# Diseases of the Uterus

**Published:** 1837

**Authors:** 


### Art. VI.—Diseases of the Uterus.

*Treatise on the Functional and Organic Diseases of the Uterus.* From the French of F. Duparcque, Docteur in Medicine de la Faculte, &c. &c. Paris. Translated, with notes, by Joseph Warrington, M. D., of Philadelphia. 1837. 8vo. pp. 455.

(concluded from the last number.)

The theory maintained by our author, in regard to the formation and development of malignant tumors, excludes, of course, all ideas of the conversion of the normal tissues into the substances which constitute the diseased structure. The diminution of the natural tissues, which is observable in cases of schirrous deposition, is the result of absorption, brought about by the mechanical pressure of the morbid mass. It is, in truth, a genuine atrophy, and not at all explicable upon the principle of a pathological transformation affecting these tissues. The only *changes* or *alterations* which take place, are to be sought in the elements which compose the *abnormal structure*. But these we shall not trespass upon the time of our readers to develope. Softening, suppuration and ulceration are phenomena, in the progress of malignant tumors, which are too familiar to the profession to require a single remark, for the purpose of elucidating their course, relations or peculiarities. A more interesting and important question, in regard to these tumors, is, whether they are susceptible of resolution? M. Duparcque decides in the affirmative, maintaining, that "all the accidental tissues are subject to the double movement of composition and decomposition." In illustration and support of this opinion, he has given two very striking cases; in each of which an e[illegible]nal schirrus corresponded most remarkably with the condition of the general system; the changes in the increase and diminution of the local disease, vibrating most exactly with the fluctuations of the individual from a state of embonpoint to one of emaciation, and vice versa.

In regard to the power of the absorbent system over the engorgements of the uterus, M. Duparcque has embodied his opinions in the following propositions:

"From the character of the matter which constitutes œdema, we should suppose (he says) it might be absorbed.

"The resolution of sanguineous congestive engorgements, or those caused by acute or chronic inflammation, is not less easy; and we may also calculate upon the resolution of indurated engorgements.

"It is, however, impossible to effect the resolution of schirrous or cerebriform engorgements, when the matters of which they are constituted are *encysted.* Under other circumstances, resolution can be accomplished more easily, perhaps, in the schirrous alterations than in others.

"Finally, those alterations which are yet incomplete, as long as the organic plexus, or net work, remains unimpaired, are more easily absorbed than those in a complete state of ramollisement."

Concurring in these views generally, we cannot yet forbear to remark, that the idea of *encysted schirrus* is new to us. We do not believe that it has any existence in pathology; neither are we prepared to admit, with our author, (upon the presumption that he is correctly translated) that schirrous tumors are more susceptible of resolution than others which are the product of common inflammation. That they are occasionally absorbed cannot be denied, without discrediting the testimony of some of the highest authorities in the profession, along with M. Duparcque himself, as well as the evidence of our own observation; but that this is a frequent occurrence, or one *easily* brought about, is a gratuitous assumption, and contrary, we think, to medical experience.

On the importance of exploring the uterus for the purpose of attaining a more correct diagnosis of its diseases, there can be but one opinion among medical men. It is, nevertheless, a fact, that this mode of investigation is almost entirely overlooked by country physicians, and much too seldom resorted to in city practice. Exposed to an almost infinite variety of pathological states, resembling each other in a large concourse of *common* phenomena, and possessing but few that are distinctive, or pathognomonic, the uterus should be carefully examined *in every case*, when there is the least ground to suppose that it is the seat of a morbid alteration. It is only by bringing the results of symptomatology to the test of one or more of our senses, that we can arrive at such a sure diagnosis, as shall constibute the basis of a rational and enlightened practice. The uterus must be recognized and *spoken of* as an integral part of the economy, in our professional intercourse with the *fair sex*, and it must be explored with the utmost freedom, (but, at the same time, with the most scrupulous delicacy,) if we would escape the imputation of empiricism, or discharge our responsible duties in a way that shall satisfy our consciences, and the just expectations of our patients. To flounder amidst the mazes of obscurity, or to surrender one's self up to the leadings of a dogmatism which never doubts, is the inevitable tendency of a system which neglects, whether from indolence or fastidiousness, the obvious methods of pathological investigation. A just regard for professional reputation, and the sacred obligations of humanity, call upon the physician, under all circumstances, and at all times, to exercise much care and caution in forming opinions which are to influence his practice; but in a class of affections so peculiarly liable to be confounded as are uterine diseases, it is an imperative duty to neglect no means that may enlighten the judgment, or direct our *methodus medendi* in the way of safety and success. Of these means, the examination technically denominated "touching" is the most important, and almost the only one employed in this country. We shall not enter into any details concerning it. Directions for its conduct are to be met with in all the standard works on midwifery, and many useful hints are contained in the volume under review. It is sufficient for us to remark, that the uterus is accessible directly through the vagina, and indirectly through the rectum, and in certain conditions through the anterior parietes of the abdomen. In general, the "vaginal touch" is all that is necessary; but in cases of obscurity, a combination of all these methods is occasionally requisite to dispel doubt. Nay, more, in France, and on the continent of Europe generally, the sight is often invoked to confirm or correct the evidence furnished by the touch. By means of a speculum and a strong light, the mouth and neck of the uterus are rendered visible, and the pathological condition of these parts is made obvious to the eye. That this method of exploring the uterus will ever become *common* in the United States we very much doubt; but, fortunately, the occasion for its employment, in our opinion, can seldom occur. When it does, gentle perseverance on the part of the physician, aided by good sense on the part of his patient, will generally prevail over that innate modesty, which is the brightest jewel of the female character, and which no man should wantonly or unnecessarily offend. For the purpose of applying leeches or medicines to the *as timœ* and *cervix uteri*, the speculum is a convenient instrument, and in this view, we apprehend, is much more important, than as a diagnostic means; in which capacity it might be discarded, as we have already intimated, without much prejudice to the cause of humanity, or of science.

The second part of M. Duparcque's work is more practical, but, at the same time, more prolix; in consequence, chiefly, of the introduction of cases to illustrate the effects of his remedies, or the progress of disease. In our attempt to analyze his book further, we propose to pass by details, and to confine ourselves principally to his therapeutics, interspersing, occasionally, such an account of the pathology and morbid appearances, as may be necessary to exhibit a faithful, but concise, picture of his labors. Our author comprehends all organic affections of the uterus under the general terms of engorgement and ulceration. The former he uses to designate a morbid "augmentation of volume in the parietes of the uterus," as distinguished from that increase which is the consequence of the development of its cavity. It is not, however, every increase in the parietes of the uterus which deserves the appellation of engorgement. The augmented volume may be connected with various pathological states of the organ. For example, the increase may be *extrinsic*, constituting *excrescences*, or interstitial, constituting engorgements, properly so called.

Of excrescences, our author mentions a variety, which not unfrequently attacks the *cervix uteri*. These are fleshy, and vary in form, size and consistence. Their development is unusually slow, and their presence occasions no particular disturbance. To this point, we have the concurring testimony of Portal, who found them in the dead bodies of many women, in whom there was no suspicion of their existence during life. In the greatest number of cases, these vegetations are soft, insensible, more or less bloody, and accompanied by a sero-mucous discharge; which does not, however, impair the functions of the organ. At other times, they acquire a very large size, and a considerable hardness of texture, and are attended with an abundant sero-muco-bloody discharge, and menstrual derangement. In this state, they are liable to be taken for carcinoma, and, according to Breschet, the mistake has been committed.

Of engorgements, properly so termed, it may be said that they affect the neck, body, or whole of the uterus at once. But the neck is the most common seat of these affections; the body being generally involved secondarily, by a progressive extension of the disease.

Engorgements are characteristic of various pathological states. They may be connected with, 1st, Hypertrophy; 2d, Œdema; 3d, Sanguine Congestion; 4th, Inflammation, acute or chronic, and induration; 5th, Schirrus; 6th, Tubercles; 7th, Cerebriform alterations; 8th, Melanic alterations."

Hypertrophy being merely an excessive development of the natural parenchymatous structure of the uterus, does not deserve to be regarded as a diseased state; and, indeed, it partakes so little of this character, that it never interrupts the functions of the organ, or gives rise to any peculiar morbid phenomena. It only deserves notice, because it is liable to be mistaken for other organic alterations, or to produce a predisposition to them. Hypertrophy of the uterus is general or partial. The former affects the entire viscus, and is very rare; the latter the neck only, and is quite common. Partial hypertrophy is sometimes an impediment to labor. In this case, it is evident that the obstacle arises from an unequal distribution of the contractile fibre of the uterus, which prevents its mouth from yielding to the expulsive efforts of the organ. The circular and longitudinal fibres being no longer in harmony, it is necessary that their relative forces should be restored, in order that the parturient action may progress regularly. The application of the ointment or of the extract of belladona, by paralysing the action of the circular fibres, is said to accomplish this end at once, and to produce in these cases a happy and speedy influence upon the termination of labor. In France, and on the continent of Europe, this practice is general, and highly extolled. In our own country, but little reliance is placed upon it. This, perhaps, proceeds from the want of care and accuracy in discriminating the cases to which it is adapted. In cases of rigidity of the os uteri, dependent upon serous or sanguine engorgement, or schirrous induration, it is evident that no benefit can be expected from the remedy.

Odematous engorgement of the neck of the uterus rarely presents itself except during labor, and in the puerperal state. It does, however, sometimes occur, independent of parturition, and is then, for the most part, connected with a leuco-phlegmatic temperament, and a broken state of health, indicated by debility, paleness, and general œdema. Its anatomical characters, as contradistinguished from inflammatory engorgement with induration, are its lightness, transparency and softness, retaining the impression of the finger when strongly pressed upon. In addition to such general remedies as appear to be indicated by the state of the constitution, the treatment resolves itself into scarifications, leeching and tonic or astringent injections. Of that form of the disease which is consequent upon parturition, the cure is spontaneous, requiring only rest and a little patience.

Of *sanguine engorgements* of the uterus, our author makes three species:—1, Engorgements by simple sanguine congestion; 2, Engorgements by congestion, with hemorrhage; 3, Inflammatory engorgements.

Simple sanguine engorgement of the uterus is the result of various determining causes; and it consists in the accumulation of a greater quantity of blood in the vascular system, or parenchymatous structure of the organ, than usually circulates in it. This plethora does not always constitute a state of disease. It exists, for example, at every menstrual period, and is then a part of a genuine physiological action. It is also observable, after parturition, and is essential to gestation. When it occurs, however, at any other periods than "those normal epochs, which we have just mentioned," or when it transcends certain limits in intensity or duration, it becomes a genuine pathological state, and demands attention.

Every congestion of the uterus consists of two stages—an active, during which the blood is determined by a fluxionary movement to the organ, in greater quantity than usual; and a passive, in which the blood, being already accumulated in its tissue, is retained there. The function of innervation lies at the very source of the "fluxionary movement;" and it is for this reason, that electrical currents, passed in the course of the nerves of the uterus, from their commencement to their termination, have been found so useful in amenorrhœa dependent upon uterine inertia, and in that form of the disease which is accompanied by vicarious hemorrhage.

An interesting question in regard to abnormal uterine fluxes would seem here to present itself; viz: whether they might not be arrested by passing electrical or galvanic currents in a direction the reverse of those employed to excite them? Do not moral emotions, which, occurring at the approach of the menstrual period, or during the flow, prevent or arrest the discharge, act after this manner?

Congestive engorgement of the uterus, besides its spontaneous development at the menstrual periods, and after delivery, is produced by numerous causes, which may be arrayed into two classes. The first acts, primarily, by exciting the "fluxionary movement;" such are violent moral emotions, or exercise, and general stimulants; to which we may add, certain emmenagogues, and the excitants proper to the genital organs, as coition and masturbation. The second comprehends such causes as operate upon the exhalent vessels of the uterus, to prevent that local depletion, which, in normal cases, is the crisis of the congestion. It is thus, for example, that cold, lively moral impressions, and the intemperate use of astringents, act during menstruation, and subsequent to accouchment. In the first instance, the *menses* are suppressed; in the latter, the *lochia;* while in both, the persistance of the fluxion co-operates to continue or increase the congestion.

The tendency of the engorgements which present at the menstrual period, and subsequent to accouchment, is to enlarge, if they be not relieved by the occurrence of the discharges, which are usual at those epochs. It nevertheless happens, in the former instance, that many repetitions of the menstrual molimen are requisite, before any permanent congestion takes place; the blood which is urged into the uterus, by the fluxionary movement, readily passing into the general circulating system, upon the failure of this primary condition of menstruation. Of this spontaneous resolution of uterine plethora, we have frequent examples in the phenomena immediately antecedent to puberty. There is at this time a periodical fluxion to the uterus, with such a condition of the exhalent, or secretory apparatus, as prevents the escape of the fluid, which is destined, in the economy of nature, to unload the vessels. Yet no disease ensues, until, by repeated sanguine determinations, without equivalent discharge, the organ becomes the seat of a permanent congestion. We have already remarked upon this state, as giving rise to displacements of the uterus, to ammenorrhœa and sterility; and thrown out some hints for the management of these affections, when they depend upon this cause. But the congestion itself demands a treatment, even when it is unattended by any of these inconveniences; for it is apt, as we have seen, to terminate in inflammation, and the most serious organic alterations. The first and leading indication in the cure, is to arrest the fluxionary movement, by removing the causes which produce or maintain it, or to determine it to other parts by derivatives, such as blood-letting, general and topical, emetics and irritants, applied more or less remotely from the seat of the congestion. Should these fail, we have then to consider the means which act more directly to produce a resolution of the engorgement. Of these, blood-letting, again, is one of the most efficient; and, in connection with cathartics, antimonials, baths and emollients, it seldom fails to produce a prompt disgorgement of the organ, or to place it in a condition to emit the menstrual fluid, which, in certain stages of existence, is the natural cure of the congestion. In many cases of the retention of this discharge, where there is reason to believe that it is owing to a spasmodic action of the exhalent apparatus indicated by pain and uterine colic, or tenesmus, the happiest effects are often experienced, by associating, with the remedies first named, sedatives and anti-spasmodics. Finally, we may substitute for the evacuation, the detraction of blood, by leeches applied upon the engorged part.

By the judicious combination of these means, we shall generally succeed in relieving *simple congestive engorgement* of the uterus; but there is, according to M. Duparcque, another species of this disease, accompanied with hemorrhage, which demands a modification of treatment, and which will now claim a few passing remarks.

This species of engorgement is developed after the same manner as that whose history we have just dismissed, and is obedient to the same causes:—

"Like it, it also results from a fluxionary movement, but excessive in degree, and particularly prolonged in duration: like it, it also consists in the penetration of a super-abundance of blood into the uterine tissue; but it differs from the above in this, that it is constantly accompanied by a sanguine discharge, which, notwithstanding its abundance and persistence, does not at all diminish the congestion: on the contrary, this engorgement tends, in spite of this spontaneous means of disgorgement, to progress and undergo particular alterations, which we will endeavor to exhibit."

There are three periods or stages in hemorrhagic engorgements of the uterus.

The type of the first period is indicated by the hæmorrhagic congestion of the menstrual periods, or better still, by those which succeed to parturition. The local and general effects of the engorgements are the same as in the case of the simple congestive state, except that there is here, more sanguine discharge—also, a more or less considerable increase of volume of the whole uterus, or of its neck exclusively; more or less deep red color; and the uterine orifice enlarged in proportion to the engorgement. The consistence of the uterine tumour may readily appear, not changed—but in general, there is rather softening, much more marked as we approach the centre of the tumour.

The hæmorrhage presents paroxysms, sometimes alarming, and which occur spontaneously, or are the result of the action of all the causes capable of hastening the fluxionary movement, or of impressing a mechanical shock upon the organ. Thus the erect position prolonged—the agitation of running, dancing, coughing—the efforts at defecation, coition and the *touch*, increase the discharge, or incite it, if it had been momentarily suspended. The engorgement increases at length, but without ever acquiring a considerable volume, as it does in cases of congestion, without hæmorrhage; and at the same time, the ramollissement becomes more and more marked; the neck of the uterus appears to be of a deep red color, appearing as though the blood had been strained through the surface of the organ, by compression. This is the second period, or second degree of the disease.

Thus far, the general phenomena evince only the loss of blood, and are in relation in intensity, with the abundance, duration, or more or less frequent repetition of hæmorrhagies. Thus, there is a progressive discoloration of the tissues, loss of strength, sense of dragging and languishment in the gastric and precordial regions, loss of appetite, or insatiable hunger, as if nature was striving by an abundant alimentation to make reparation of the vivifying fluid, incessantly lost.

Let us repeat the essential characters of this kind of engorgement; tumefaction, softening, more or less deep red color of the cervix uteri, discharges by the orifice, of blood variable in quality, and an exudation, often appreciable of an analagous fluid through the surface of the tumour.

These discharges are excited or aggravated by the *touch* and pressure.

These pathognomonic signs are recognised directly by the *touch*, and the use of the speculum.

At length, the third period arrives; sometimes after a very short interval, at others, when the affection has continued during several years, in the first and second degrees. A new condition of things has obtained, when the third period occurs.

As this alteration is commonly extended to the neck of the uterus, or affects that part only, we obtain by the touch and the speculum, the signs which are proper to it, and which characterise it. We find then, at the bottom of the vagina, and advancing more or less into that cavity, a tumor formed by the engorged neck of the uterus, of a brown red colour, its surface, which appears pretty smooth to the sight, being always covered with layers of coagulated blood, seems a little uneven to the touch. By pressing this tumor, a distinct sensation of crepitation is produced, depending probably upon a displacement of the half coagulated blood, which infiltrates the diseased tissue; at the same time that we very evidently observe this dark fluid escaping from the surface of these tumors, as if it was expressed from a sponge.

At the opening of dead bodies, we find the altered part puffed, of a dark color, soft, friable, and pulpous. The uterine parenchyma is reduced into a mass of fibro-cellular and vascular filaments, easily torn and lost in the midst of the dark and coagulated blood which is infiltrated into it. In a word, this alteration presents an exact resemblance to the tissue of an engorged and half putrified spleen. This alteration appears to progress from the internal surface of the uterus to the external, in which we commonly find a more or less thick layer of uterine tissue, still unaltered.

This alteration is often found in the midst of parts presenting traces of inflammation, surrounded by purulent cavities. The altered mass is itself strewed with small cavities, and infiltrated with pus mixed with black blood. How is this transition affected from the engorgement of a simple congestive state, in which the blood merely occludes the capillary system, and which constitutes the first degree of alteration, to the second or third degrees in which the blood indurates the meshes and interstices of the uterine parenchyma?

Is it not that the prolonged contact of the fluids has weakened the tissues, or as may be said, macerated and destroyed them, with the exception of some part of the cellular and capillary tissue? The natural or accidental state of relaxation, or of atony of the uterine parenchyma, should, if this be the case, favor the development and transformations of this kind of disease;—this is, in fact, what observation proves. We see the hæmorrhagic engorgements affect, in preference, women of a soft constitution, and lymphatic temperament; those in whom the uterus has been fatigued by numerous and laborious accouchements; finally, those in whom the menses are remarkable for their habitual abundance, the length of their duration at each period, and the frequency of their return.

At the "turn of life," this affection is favored by these two conditions: 1st. The return or the unusual perseverance of fluxionary movement: 2dly. The loss of elasticity, the organic feebleness of the uterine parenchyma, which permits it to be distended and infiltrated by the fluids and the blood which the fluxionary movement urges thither.

Before reaching the third period, the hæmorrhagic engorgement, in a certain number of women, takes another movement or direction. The centre of alteration, represented by the orifice and internal surface of the uterus, soft, macerated and destroyed, is transformed into an ulcer presenting a stratum, more or less thick, soft and putrilaginous; the limits of which, marked by chronic inflammation, form an almost scirrhous base. The general symptoms also disclose, by their intensity, or their special character, the existence of hæmorrhagic engorgement, arrived at the last degree.

To the general and complete discoloration, arising from excessive discharges, is joined a straw-like yellow tint, as in ordinary cancerous affections. The eyes are dull, the feebleness is extreme; and notwithstanding women, who, in common, have much embonpoint, still preserve the appearance of it, a firm flesh has given place to a general bloating, which conceals the marasmus of the muscular portion, and the flesh is really soft and flabby.

Hemorrhagic engorgement of the uterus is always a very serious disease; but when it has arrived at its third state, necessarily fatal. There is then such a destruction of parts as to render a return to the normal state of the tissue affected impracticable. But in the first and second stages of the disease, the danger from disorganization is subordinate to that which arises from hemorrhage; and there is good reason to anticipate recovery from the adoption of a judicious and rational treatment; which none of our readers, who have been attentive to the principles we have endeavored briefly to exhibit, can be at a loss to comprehend.

The primary indication in this engorgement, and the means of fulfilling it, are the same as in the preceding species. But in addition to the remedies there indicated, for the purpose of arresting or changing the local determination which maintains the congestion, we are called upon to adopt others, with a special reference to the hemorrhage, which, by its continuance or violence, constitutes, in many instances, the immediate source of danger. Among the anti-hemorrhagic remedies, astringents justly occupy the first rank. But in order that they may produce any beneficial result, they must be adapted *accurately* to the pathological state. If the physician, occupied with a mere incidental phenomenon (*hemorrhage*) overlooks the fundamental disease, and resorts to astringents, with a view of restraining the flow before the local determination, which maintains it, is arrested, he will infallibly perpetrate mischief. He may secure, it is true, the object of his *limited* therapeutics—*he may restrain the hemorrhage;* but he will inevitably precipitate his patient into greater peril, by aggravating the engorgement, or converting it into a state of inflammation—far more dreadful in its consequences than the primary malady. On this subject, M. Duparcque has given us some very instructive lessons, and we might appeal to the experience of every physician, at all conversant with uterine diseases, for parallel cases, to attest the mischievous results of a *premature* administration of astringents under the condition here stated. There is, nevertheless, a point in hemorrhagic engorgements, where this class of medicines will be found to exert the most favorable effect; but this point does not consist with a persistance of the "fluxionary movement," or an active state of the capillary circulation of the uterus. It is only after the local determination has been subdued by appropriate treatment, or the uterine tissue has fallen into a state of atony, denoted by the long duration or violence of the sanguine discharge, terminating in general debility, that astringents are indicated. Under these circumstances, the most auspicious results may be anticipated from their employment.

It is in the same relaxed state of the uterine fibre, that ergot has acquired a reputation in the treatment of hemorrhagic engorgement. Nor is it difficult to comprehend its *modus agendi*. By its power over the contractility of the uterus, its tissue is condensed with the effect of throwing back the engorged fluids into the current of the circulation, and of placing the organ in a condition to resist their return. This explanation of the action of ergot is consonant to its general properties; but after all, it may not comprehend the whole secret of its *almost miraculous* power in this form of uterine hemorrhage. M. Duparcque claims for it an astringent property, and does not hesitate to aver, that he has arrested, by its use, hemorrhage from other organs besides the uterus. In illustration of the views and practice which we have endeavored to present in a condensed way, our author gives fourteen interesting cases, for which, as well as the diagnosis between hemorrhagic engorgement in the third degree and other profound organic alterations, we refer our readers to his book.

In regard to inflammatory engorgement of the uterus, or metritis, our author adopts the common division of acute and chronic. In his account of the etiology and symptomatology of the acute form there is nothing that is peculiar. His history of the affection is essentially the same as that of other writers; and a similar observation may be predicated of his treatment, with one or two exceptions. Based upon the character of the disease, it comprehends the whole circle of anti-phlogistic remedies. Bleeding, general and topical, proportioned to the intensity of the phlogosis, evacuants, antimonials, diaphoreties, revulsives, irritants applied on different parts of the body, and emollient cataplasms to the hypogastrium, with absolute repose and rigid regimen form an aggregate of remedies sufficiently consonant to the general routine of practice, to exempt us from the necessity of any thing more than a simple allusion to this portion of the book. The only peculiarities in the treatment recommended by M. Duparcque are antimonial inunctions, and the application of leeches upon the engorged part.

Of the value of tartarized antimony, given by the stomach, in the subdued forms of inflammatory action, the profession has been long apprized; but this favorable estimate of the medicine has been much enhanced of late years, by the remarkable effects which are observed to result from its use, in the whole class of phlegmasia, when employed in large doses, after the counter-irritant plan of the Italian physicians. That it is capable, however, of affecting the constitution, and of producing the same beneficial results by *endermic administration*, was a revelation reserved for M. Duparcque, who has given several cases to prove the efficacy of this method of employing the remedy. The unguent, which he uses, is composed of one drachm of tartar emetic and one ounce of simple cerate. Of this, half a drachm, rubbed into the inside of the arms or thighs, is sufficient for a single application. The only condition of its use, is that the violence of the inflammation shall have been previously abated, by the employment of the antiphlogistic means we have pointed out under this head. The prompt dispersion, by this method, of inflammatory engorgements of the uterus, which, having attained a certain point in the progress of resolution, are, nevertheless, stationary under the ordinary modes of treatment, has impressed M. Duparcque with the greatest confidence in its powers, under these circumstances. Nor is he less enamored with the effects, in these cases, of *local bleeding, made immediately on the diseased organ;* for he avers, (and we are disposed to assent readily to his assertion) that "it is neither less prompt nor less efficacious than antimony employed in frictions." Of this remedy, in engorgements of the uterus, we shall speak more fully in a subsequent part of this review.

The diagnosis between induration of the neck of the uterus and schirrus, has always appeared to us to be a matter of great difficulty; and the attempts to establish it upon a consideration of the form, consistence and color of these tumors, or the character of the pains which attend them, are exceedingly infelicitous and unsatisfactory. It is not, therefore, in our opinion, without good reason that M. Duparcque has associated and treated of chronic metritis, induration and schirrus, under the generic appellation of *hard engorgements of the uterus.*

The ANATOMICAL CHARACTERS of *schirrus* and *induration* are thus pourtrayed by our author:—

"Schirrus is characterized by a white color, with a bluish or grey tint of its tissue, which is semi-transparent, very hard, resisting, creaking under the instrument which divides it; according to the alveolar or radiant disposition of its net work, and its more or less white color, it presents the aspect of a chesnut, a turnip, or the intervertebral cartilages.

Ordinarily, little, or even no traces of the tissure proper to the organ, are found in its composition.

In the engorgement by induration, the tissue which composes it, and which presents, *in the living subject*, a hardness analagous to that of schirrus, is, if not *soft*, at least *pliable;* the color of the tissue, which is the seat of it, has only grown pale. We can still readily enough distinguish the tissue (*normal*) the fibres of which are only dispersed by the presence of a more or less concrete fibro-cartilaginous matter, which we can sometimes indent by pressure or scraping, especially after maceration for some days. It would seem that here, there was merely a mixture of the product of chronic inflammation with the tissue of the organ, whilst in schirrus, there is a sort of combination, which gives to the alteration a more homogeneous aspect. In proportion as the induration is of longer standing, the anatomical differences which distinguish it from schirrus, diminish and become effaced—the tissue of the organ sinks down and disappears, the infiltrated matter becomes more and more concrete, and in passing into a species of cartilage, it acquires a greyer color, and a more transparent tint, characteristics of the schirrous state."

It appears, from the above description, that these two pathological states—*induration* and *schirrus*—are so assimilated, that even the scalpel fails, in many instances, to detect any essential difference between them; but even granting that the anatomical characters of these alterations were more distinct, of what avail would this circumstance be to us, in a practical point of view, unless they presented also well marked diagnostic signs in the living subject; but this they do not; and we are left, in making up our opinion as to the particular character of the alteration, to the uncertain guidance of a few circumstances connected with its history. An extensive comparison of cases has furnished M. Duparcque with the following results:

1st. The engorgements which affect the uterus of *girls*, are, in general, inflammatory. They, in this case, usually, though not always, implicate the *whole* organ.

2d. The same is true of those engorgements which succeed parturition, at or before term; but contrary to what takes place in the preceding case, the *neck* of the uterus is commonly the *exclusive seat* of the alteration.

3d. The hard engorgements of the uterus, which occur in young females, whatever be their seat or their cause, belong much more frequently to the *indurated*, than the schirrous state.

4th. These engorgements may preserve their character of chronic metritis or of simple induration for many years; but at the approach of the *critical age*, they have a tendency to pass through the schirrous state to confirmed cancer. But if they pass this period without changing their nature, they then undergo transformations both cartilaginous and osseous.

5th. The engorgements which occur during the critical age, are, in general, and at the onset, of a schirrous, cerebriform nature, and when they commence by an inflammatory state, this is only transitory, and endures but a short time.

6th. The engorgements which arise, and are developed a certain time after the normal cessation of menstruation, (which, it may be said, happens very rarely,) present an extremely compact schirrous tissue.

7th. The engorgements which promptly acquire a certain volume, belong rather to chronic metritis, than to schirrus, of which the development is, in general, more slow and gradual.

From a review of the whole ground in relation to the *special* diagnosis of these two states, (induration and schirrus) we are led, irresistably, to the conclusion, that the evidence which must guide us in pronouncing, in the living subject, upon the existence of either, to the exclusion of the other, is uncertain and conjectural. But in relation to the general characters of hard engorgements, or the phenomena which distinguish them as a class, we are by no means left in the same doubt and perplexity. And this, fortunately, is sufficient for every practical purpose; the basis of the treatment being the same, whether the alteration consist in chronic metritis, induration or schirrus. M. Duparcque lays down three indications to guide us in the management of hard engorgements of the uterus.

"1st. To dissipate, or remove from the diseased organs the material elements of the alteration.

"2d. To modify or destroy the exaggeration of the secretory or nutritive functions, by which these elements are separated from the blood, and assimilated to the affected organ.

"3d. To excite or favor the absorption of the deposited morbid matter."

We do not deem it necessary to enter into any details concerning the means which he proposes to fulfil these objects. We write for the *initiated*, and of course shall not wilfully expose ourselves to the imputation of being tedious, by the recital of matters that are known to every member of the profession; but shall confine ourselves strictly to such points as, from their novelty or importance, are invested with peculiar interest. Bleeding, general and topical, evacuants, derivatives and abstinence are the great instruments wielded by the profession for the accomplishment of the first indication proposed by our author; and we do not perceive that he has added any thing to the stock of our knowledge in this respect, except in the single suggestion, (important we admit,) of abstracting blood immediately from the diseased organ. From this he affirms, that he has

"Obtained the most happy effects in cases of engorgements, either of the body or of the neck of the organ, even when he had suspected them to be of a *scirrhous* nature, and when they had already resisted the treatment ordinarily indicated in this dreadful affection. The rapidity with which the resolution is effected by this kind of application, is such, in certain cases, that it is necessary to have the evidence of it—not to be tempted to accuse of prepossession or of exaggeration, him who should announce such facts.

The first effect of the application of leeches to the neck of the uterus, an effect which uniformly occurs, even in cases which do not admit of cure, or in confirmed cancer itself, is to calm the sacro-lumbar distress, the lancinations, and indeed all the severe pains which are the usual attendants on profound alterations of the uterus.

The number of leeches should be proportioned to the volume of the engorgement, the degree of predominancy of the inflammatory symptoms, and to the general state of the strength. It may, however, be remarked, that this *direct bleeding* produces less feebleness, other things being equal, than general bleeding. It may also be used in those cases where the latter might be prejudicial; as for example, in the last stage of cancer.

Let us observe, that in general, the most insupportable disturbances in the cancerous affections of the uterus, are less the result of the cancers themselves, than of the inflammation which they either determine to, or develope in that part.

Further,—The schirri, like the soft encephaloid masses, become opened into ulcers only after inflammation is developed in them, as is proved by the phenomena which accompany this effort of elimination, and the matters which escape from them. It is particularly at that time, that those excruciating pains are manifested, which characterise the progress of cancer, or disclose their existence, when till then they have been unperceived. Now, in these cases, the leeches applied upon the tumors, arrest inflammation, suspend the pains which it had occasioned or exasperated; and may also retard the march of the disease, and delay the period of its fatal termination.

In cases of cancerous ulcer, it is also to be observed, that the disease involves the neighboring parts only, after inflammation has been developed there, in the form of protuberances or engorgements; the red color and the painful sensibility of which, attest its inflammatory nature.

In these cases also, leeches applied upon these parts, arrest inflammation, and consequently allay the pains which it produced, as well as suspend the progressive march of the ulcer.

Thus, therefore, leeching upon the disease itself, effects its cure, or furnishes one of the most powerful palliations which we possess in the incurable alterations of the uterus.

It is not only when the engorgement occupies the neck of the uterus, that they are useful, for they do not produce less good in the engorgements of the uterus itself.

When the uterus is low, and when in separating the labia externa its neck can be seen, a direct application of leeches can be made *upon it.* Accident once furnished me the opportunity of making this application without my wish to do so, and not without some uneasiness as to the consequences which might result. I did not then appreciate the advantages to be derived in practice from the happy and unexpected results which it produced. It was reserved to the practical sagacity of M. Recamier, to foresee the advantages which could be obtained from these valuable means.

In other cases, when the uterus occupies its normal situation, the speculum serves to expose its neck, and conduct the leeches to this part.

When the instrument has been placed, so that the circumference of its opening exactly and exclusively embraces the neck of the uterus, to prevent the leeches from biting any other parts, a quantity of water should be injected, to wash away any matters which, by their qualities, would hinder the leeches from taking hold.

They are then to be placed in the tube, pushed up and maintained against the neck, by thrusting in after them, a cork or tampon of linen, to prevent their return; the tampon is to be withdrawn as soon as the leeches attach themselves to the uterus, which they commonly do very promptly.

To the great astonishment of the patients, the biting and the suction of these animals are scarcely felt; sometimes, however, the patients, during this little operation, which they very much dread, because of the habitually painful state of the diseased uterus, feel a tickling, heat, and quick lancinating sensation in the pelvic and sacral regions. All these sensations are easily supportable. In ten or twelve minutes, the leeches have taken, filled and fallen off; they slide out of the speculum, or may be removed by the finger or forceps. After having washed the parts by warm water injections, the speculum may be withdrawn.

It should be remarked, that when the engorgement appears to be of a scirrhous nature, by its hardness, the length of time it has existed, and the whiteness of the tumour; the punctures bleed very little after the leeches fall off, and that when on the contrary, the neck of the uterus was colored, of a moderate hardness; in a word, when the engorgement was more inflammatory than indurated, the bleeding was more abundant, and might even become alarming. It is however easy to suppress the hæmorrhage by the tampon. The sanguineous discharges become more and more abundant, almost constantly, in proportion as the first applications of leeches, in bringing the uterine tissue to its natural anatomical state, render it more permeable to the blood.

Regard must be had to all these circumstances, in determining upon the number of leeches to be applied. We have not used more than a dozen at a time; six to eight most commonly are sufficient to produce a bleeding free enough, and in the subsequent applications the number may be diminished.

It has been said, that the orifices made by the leeches upon the neck of the engorged uterus, would not cicatrize, that they remained open without always becoming ulcerated. It has been impossible for me to detect any traces of the bites of the first leeches, when I have subsequently repeated the applications; but this may readily result from the retraction of the engorged part, which necessarily brings about a proportionate diminution in the extent of the punctures, as we see in profound incisions in the tumefied part, the tongue for instance, become reduced into linear divisions, imperceptible after the disgorgement.

The unlooked-for success which I have obtained from the application of leeches upon the neck of the uterus, in cases of engorgement, no less than in the most profound and most advanced alterations of the uterus, authorizes me to place this means at the head of all those which have been in repute against these formidable affections. Cure of the one, and relief of the others, have been in great part owing to this means. Other therapeutic measures ought not to be neglected; for they in general add to the efficacy of this, and in some cases produce the advantageous effects that could not be obtained from local bleeding.

Nevertheless, we should have recourse to the application of leeches to the neck of the uterus only, after having practiced one or two general or derivative bleedings, in order to prevent the consecutive congestions, which would render the employment of this means, more prejudicial than useful.

Of the practice described in the preceding extract, we have no experience, nor do we believe that it has been extensively tried in the United States, much as our physicians are in the habit of resorting to the topical abstraction of blood, in local inflammatory affections. Analogy, aside from all experience, would induce us to place upon it the most favorable estimate. But as has been justly remarked by our author, it should not be allowed to take the place of general bleeding, which, in a great majority of cases of hard engorgement of the uterus, is an essentially curative remedy, and in all, an important preparatory means.

Among the remedies which are calculated to fulfil the second indication of our author, cicuta has acquired a very high reputation, and indeed by many it has been regarded as a specific in cancerous affections. But it seems doubtful whether, after all that has been said in praise of it, it possesses any directly resolvent property. Its action is very probably limited to the nervous systym, which we know holds an unlimited control over the movements of the blood, which supplies the material of the morbid alteration, as well as over the absorbent vessels which are to remove it. However this may be, cicuta is, according to M. Duparcque, of all the substances which belong to the modifiers of nervous influence, the article from which the most advantageous effects have been obtained in schirrous and cancerous affections. The preparation used by M. Duparcque is the extract, prepared in vinegar or alcohol. It is given in pills, in doses of two grains a day, which may be increased gradually to a drachm in the same period. Neither the acetous nor alcoholic extract of cicuta disturbs the stomach as other extracts are apt to do; but they impart to it tone, and re-establish the digestion, which is so frequently impaired in these severe affections.

Aconite, belladonna, hyosciamus, solanum and lactucareum have each been invested, by their partial advocates, with a similar specific power over the profound organic alterations of the uterus; but there seems to be no reason for conceding to those articles, what has been denied to their more important associate. The truth is, that they are all useful as sedatives, not only to relieve pain, which is so prominent a symptom in these diseases, but to reduce the increased innervation of the part to a state which shall admit of the resumption of its physiological functions, absorption and secretion.

As a narcotic, to relieve pain, opium may be used, when other remedies have failed. But from its stimulant properties, and its consequent tendency to produce capillary congestion, it should be resorted to only in extreme cases. It is a good rule, in regard to the administration of narcotics, for the purpose of allaying pain in chronic diseases, to commence with small doses, and gradually to increase; and when the economy becomes accustomed to their impress, to suspend, vary, or substitute them by others. Often, very mild medicines will calm the agitations of the system, when stronger have failed. The syrup of white poppies, or the distilled water of lettuce, seldom fail, it is stated by our author, to subdue the vigilance occasioned by the "painful and distressing sensations" consequent upon organic diseases of the uterus.

As a part of the sedative medication of the diseases which attack the neck of the uterus, emollient and anodyne injections have always occupied a share of professional attention. They do good, undoubtedly, by cleansing the parts, and removing the irritation of acrid discharges; and in this way, perhaps, as well as by their action upon the local innervation, contribute at least to the comfort of the patient. "A decoction of marsh mallows, flax seed, lettuce, mullein, pellitory of the wall, groundsell, nightshade, poppies, stramonium, mandrake, cicuta,henbane, the watery solution of opium, or of its salts, and of the extractof belladona," have been all used occasionally, with results more or less favorable. In other instances, greater relief has been obtained from cold water alone, as a hip bath, vaginal injection, or enema. M. Duparcque assures us, that he knew a lady, "affected with an ulcerated cancer of the uterus, in an advanced stage, who, after having tried every sedative, found comfort to her severe sufferings only in cold water," employed in the modes designated above.

As co-operating in the same plan of treatment, the patient should be removed from every source of inquietude, and placed in a situation where she might enjoy the advantages of cheerful conversation and country air. "These alone have sometimes been found sufficient to favor a disposition to resolution of obstinate engorgements. In short, every hygienic precaution, which is capable of acting upon the nervous system, by moderating its influence, or by re-establishing the harmony of its functions, and consequently abstracting its abnormal concentration upon the affected organ, will afford important aid in the management of these deplorable affections.

We have been thus particular in laying before our readers the means of fulfilling our author's second indication, because, in our opinion, they constitute, along with rigid abstinence, the only possible hope for the eradication of an *open cancerous affection*, while they are, confessedly, amongst our most useful auxilliaries in the management of the *hard engorgements* of the uterus.

The means of fulfilling our author's third indication, that of promoting *directly* the absorption of the morbid matters which constitute the elements of the hard engorgements, are, we apprehend, very limited, if, indeed, there be any known to the profession. All the remedies, which we have detailed, are, in a certain sense, discutient and resolvent; that is to say, they promote resolution by lessening, or determining to other parts of the system, the fluids from which the diseased mass is elaborated; or they accomplish the same end by reducing the innervation of the part to its normal state, and thus enabling it to resume and carry on its proper physiological actions. But besides these *secondary* or *indirect* resolvents, the indication of our author contemplates a class of medicines, whose *primary* action is upon the absorbent system, and which are hence entitled to be regarded and designated as *direct or special resolvents.* Whether there be any such, we repeat, is very problematical; and yet consulting the opinions and testimony of medical men, we should not feel ourselves justified in denying to mercury, iodine, arsenic, tartar emetic, and other metallic salts, a special power to quicken absorption, and to remove from the affected tissue, the materials of its alteration. It does not comport with our plan, and certainly not with the limits to which we have restricted ourselves, to point out the particular pathological states, or other circumstances which should lead us to adopt one or other of these medicines. We will only observe, *en passant*, that calomel and the preparations of mercury, upon which so much reliance is placed by the profession at large, in the treatment of the hard engorgements of the uterus, have appeared to our author to produce salutary effects only in cases dependent upon a syphilitic taint. While iodine is more particularly adapted to such cases as have received a peculiar impress from the predominance of the strumous diathesis. Of arsenic, he thinks the advantages are too uncertain and contingent to justify the risk of inducing "the serious accidents which it sometimes occasions."

Judging from isolated cases, there would seem to be many other medicines endowed with a resolvent power; such are "the *cyanurate* or the *hydrocyanate of lead*, taken internally, or administered in injections or baths; the *hydrocloɪate of barytes;* the *muriate of gold;* the *sub-carbonates of lead and potass;* the *sulphates of ammonia and copper;* and the *green sulphate of iron;* the *muriates of iron and ammonia;* the *hydriodate* and the *chromate of potass;* the *borate of soda; oxygenated water*, &c. &c." In regard to the employment of these medicines in the schirrous and cancerous degenerations of the uterus, it may be truly affirmed, that the trials have been too limited to justify our reposing much confidence in them, or to enable us to deduce any principles which shall be competent to direct us in their application. One remark only it seems important to make, and that is "that they are all improper before depressing the vitality of the engorged organ, and arresting the development of the alteration, by the previous use of medicines, to fulfil the two primary indications." It is an invariable law of absorption, (from which there is, we believe, no exception,) that its activity is in an inverse ratio to that of the circulation. Hence the employment of resolvents, intended to act specifically upon the absorbent system, should always be preceded by the anti-phlogistic treatment, and in cases of local disease, by such other means as are calculated to depress the vitality of the part. When resorted to earlier, they not only fail to do good, but are positively mischievous, by increasing the engorgement.

For numerous interesting cases to illustrate the "hard engorgements of the uterus," their progress, and the effects of the various remedies which have been recommended, as well as the general results deduced by our author from the history of the whole, we must refer to his work, and proceed to bring our review to a close, by a very brief allusion to the other subjects of which it treats.

TUBERCLES occasionally attack the uterus, as they do other organs of the body. "They are either encysted or not—of a spheroidal shape—from the size of a grain of sand to that of a pea, and even of a hen's egg. The substance of which they are formed, is of a transparent grey; the consistence semi-cartilaginous, containing no vessels, and frequently exhibiting a ɪadiated appeaɪance.

"They at length become opaque and yellowish; then soft from the centre to the circumference; [*this order is not invariable, but is often reversed*] and are then transformed into a casiform matter at first, afterwards like curds and whey, homogeneous and puriform, susceptible of being absorbed; but most frequently bursting the cavities encysted or otherwise, which envelopes it. It is only when tubercles take on this last termination, that their existence can be suspected in the tissue of the uterus; even then nothing ordinarily indicates their development; their presence does not appear to restrain the menstrual function of the organ, nor consequently occasion any general disturbance."

ULCERATIONS and CANCEROUS DEGENERATIONS, in their advanced states, constitute the final topics of M. Duparcque's work. We will not, at this time, attempt to present even an epitome of his views on these interesting subjects; but content ourselves with the statement of a few deductions from his extensive experience, referring those of our readers who are curious about his opinions to the book itself, for their gratification.

"The prognosis of confirmed cancer is always extremely serious," though not always fatal. Numerous instances of spontaneous recovery, as well as experiments with medicinal substances, attest its curability, and should incite us to the employment of remedies even in the most hopeless cases. But chiefly should we feel ourselves constrained by the desperate character of those profound alterations, in their most advanced stages, to arrest, *in limina*, the engorgements which constitute their primordial seeds, and without which they are seldom developed.

It is not, therefore, without good reason, that our author has urged upon us the indispensable necessity of a timely and appropriate treatment for these simple and primitive engorgements, which being properly attended to at first, are easily relieved, but if neglected, pass into a state of incurable disease, admitting only of palliation from medicine, or of very equivocal benefit from the resources of surgery.

On the subject of the amputation of the neck of the uterus, we were gratified to find our author placing the weight and influence of his name in opposition to the rashness of those Continental surgeons, who, both by their precept and practice, have contrived to give a false eclat to this cruel and often unnecessary operation. Where the disease is limited to the neck of the uterus, this may be excised undoubtedly; and if the patient escape the perils incident to the operation, such as inflammation and fatal wounds of neighboring organs, to which we might add the consequences of the violent shock inflicted upon the nervous system, she may do well. But who, let us ask, in the present state of obstetrical practice, will presume thus to limit the pathological condition, or to say that it may not have invaded the tissue of the body of the uterus itself? in which case the operation, so far from preventing, would only accelerate a fatal catastrophe. But if there is danger of mistaking the extent, there is not less, of misapprehending the character of the disease, and consequently of having recourse to this cruel proceeding unnecessarily. An inspection of the pathological specimens which adorn the cabinets of schools, and individuals, on the Continent of Europe, would show how prevalent is this fallacy. In many cases, M. Duparcque asserts, there was not a vestige of disease to justify the use of the knife.

We have thus travelled over the subjects presented to us in M. Duparcque's treatise; and it would, we think, be unjust to deny to him the merit of *original*, *just* and *instructive* views, and of great practical acumen. But these qualities, we fear, scarcely redeem the work from the reproach of being tedious, and even repulsive, by the prolixity of its illustrations, and the detail of unnecessary cases. Retrenchment in these respects would certainly render it much more attractive, without impairing its value.

Of the *translation* we can scarcely venture to speak in the terms which it deserves, lest by those who have not seen the book, it should be supposed that we were capable of violating the *dignity* and *impartiality* of criticism, for the indulgence of a splenetic temper; but this much we are constrained to say, that of all the literary efforts of this character, which it has fallen to our lot to examine, this exceeds, in the multitude and inexcusableness of its faults. We know how difficult it is for a translator to avoid the idioms of a foreign language, and to preserve, at the same time, the character of a faithful interpreter; and we hope we are prepared to make every proper allowance for this too common fault. But in the present instance, there is such a predominance of *Gallicism*, as to be irreconcilable with a just respect for our mother tongue, and inconsistent with perspicuity, and accuracy of version. Besides this, its most conspicuous fault, the translation is deformed by errors in grammar, which are disgraceful, and mistakes in orthography, which charity would lead us to charge upon the typography, if they did not occur with a uniformity that declares them to be the offspring of ignorance. We marvel how physicians, eminent for learning or station, could prostitute their names, (as is seen in the recommendations which are affixed to this work) by an attempt to give currency to a production so crude, and calculated, in every page, to repel—American students of taste from the investigation of the interesting class of diseases of which it treats.

